# What Is Seen Is Who You Are: Are Cues in Selfie Pictures Related to Personality Characteristics?

**DOI:** 10.3389/fpsyg.2017.00082

**Published:** 2017-01-31

**Authors:** Bojan Musil, Andrej Preglej, Tadevž Ropert, Lucia Klasinc, Nenad Č. Babič

**Affiliations:** ^1^Department of Psychology, Faculty of Arts, University of MariborMaribor, Slovenia; ^2^Faculty of Civil Engineering, Construction IT Centre, University of MariborMaribor, Slovenia

**Keywords:** selfie, self-presentation, social media, selfie coding, personality assessment

## Abstract

Developments and innovation in the areas of mobile information technology, digital media and social networks foster new reflections on computer-mediated communication research, especially in the field of self-presentation. In this context, the selfie as a self-portrait photo is interesting, because as a meaningful gesture, it actively and directly relates the content of the photo to the author of the picture. From the perspective of the selfie as an image and the impression it forms, in the first part of the research we explored the distinctive characteristics of selfie pictures; moreover, from the perspective of the potential reflection of a selfie image on the personality of its author, in the second part we related the characteristics of selfie pictures to various personality constructs (e.g., Big Five personality traits narcissism and femininity-masculinity). Important aspects of selfies especially in relation to gender include the tilt of the head, the side of the face exhibited, mood and head position, later related also to the context of the selfie picture. We found no significant relations between selfie cues and personality constructs. The face-ism index was related to entitlement, and selfie availability to neuroticism.

## Introduction

Developments in mobile information technology, digital photography and social networks have stimulated the formulation of new research agendas in the field of human computer interaction, computer mediated communication and cyber-psychology. In particular, self-presentation as an aspect of behavior facet has considerable potential to interact with new communication technologies. In the context of self-presentation, the medium of photography, and in particular the self-portrait as a subtype of photography, is particularly interesting. The popularity of this kind of photography has resulted in a new word: “selfie.” This became the word of the year in 2013 (Oxford Dictionaries, 2013).

Some researchers link the popularity of the selfie to the global proliferation of mobile phones containing a camera and their integration with social networks (SNS) (Gunthert, 2014; Senft and Baym, 2015). However, the technology itself does not determine behavior; therefore, we should understand selfies as more than merely technological artifacts. Instead, selfies could be understood as a means of communication, as symbolic gestures with their own purpose (Senft and Baym, 2015). The technical understanding of a photograph as a mechanical imprint of physical reality should be replaced by its cultural form, taking into account a variety of purposes and meanings (Lister, 1995). At the same time, smart phones that have cameras and are linked to SNS represent an important context which separates the selfie from other forms of self-portraits (Tifentale and Manovich, 2015) and makes it interesting as a research topic.

### Personality, social networks, and selfie

Personality is a major predictor of human behavior in online environments (Błachnio et al., 2013; Orchard et al., 2014). Studies show significant links between the Big Five personality traits (McCrae and John, 1992) and several dimensions of SNS usage, like motivation (Orchard et al., 2014), self-monitoring (Hall and Pennington, 2013), impression management (Leary and Hoyle, 2009; Rosenberg and Egbert, 2011; Wang, 2013), communication patterns (Balmaceda et al., 2014) and social media language (Park et al., 2015). Online behavior could also be linked to personality traits known as the Dark triad—narcissism, Machiavellianism and psychopathy—and a tendency to self-objectification (Amichai-Hamburger et al., 2002; Fox and Rooney, 2015). Additionally, psychological traits could be linked to the posting of photography on the Internet (Eftekhar et al., 2014) and selfie posting behavior (Qiu et al., 2015; Weiser, 2015; Sorokowska et al., 2016).

When the selfie initially appeared on social networks, it was intuitively considered, especially in popular media, as a sign of pathology (e.g., narcissism) in SNS users. However, research does not support this intuition, and selfie posting behavior shows weak links to some specific facets of narcissism in combination with sex (Fox and Rooney, 2015; Sorokowski et al., 2015; Weiser, 2015; Barry et al., 2017). Selfie posting is recognized as normative behavior practiced by the majority of SNS users (Barry et al., 2017). At the same time, the frequency of selfies online is relatively low in comparison to the quantity of other photographs (Tifentale and Manovich, 2015).

The research summarized above mostly targets the expression and recognition of personality traits from online behavior, based on data mining techniques and on a huge amount of information. These studies count various items available online, trying to reconstruct the digital footprint of the users. Examples of such items include the number of social network posts, the number of images posted or comments received, the number of “friends” and likes received, the frequency of profile image updates, etc. Recognition of personality traits from this complex digital footprint (Hall and Pennington, 2013) or from image posting behavior (Eftekhar et al., 2014; Sorokowski et al., 2015; Sorokowska et al., 2016) proved to be possible and very accurate. Some authors claim that it could be even more accurate than reporting by close relatives or even self-reporting (Youyou et al., 2015). On the other hand, these approaches require a large amount of data from user profiles. It can also be noted that quantitative research usually omits the content of any available messages or images. A similar deficiency has also been recognized by Shelton and Skalski (2014) with regard to Facebook research in general. The studies by Eager and Dann (2016) and Qiu et al. (2015), which focus on selfie content analysis related to the self-presentation process or personality traits, are exceptions in this regard.

### Picture: analysis of the selfie as photography

Although the selfie is linked to the context of mobile devices and social networks by definition, in its basic form it remains photography. Therefore, it makes sense to consider the selfie as a photographic genre and interpret it via concepts of photographic theory, like index, composition and reflexivity (Frosh, 2015).

However, it should be noted that the selfie is not primarily an art form. In some rare cases it is indeed used as a tool for artistic expression, but in general it is a casual snapshot. The snapshot represents the most widespread type of photograph. It has not only esthetic value but also a specific social purpose (Batchen, 2008). Therefore, a selfie is a gestural image with a direct communicative purpose. It is an index showing the activity of its author, and its meaning could be interpreted as “see me showing you me” (Frosh, 2015, p. 1610).

From the above, the selfie's potential for recognition of the personality characteristics of its author could be anticipated. Frosh (2015) additionally explains this potential through identification of the selfie as a photographic genre of personal reflexivity, where attention is focused on the context and self-presence of the author. The spatial distribution of particular elements in the image, the composition, is influenced by the technology as well as by body limitations and sensorimotor coordination skills (Frosh, 2015). For this reason, the selfie could be seen as distinct in comparison to other forms of self-portraiture. The selfie is an expressive gesture by its author. Because it is not considered as an artistic expression, we should interpret its content and composition through the function it serves. The way the message is forwarded and the content of the message in selfies are both linked to the author present in the image. Therefore, we assume that a selfie, as a meaningful picture, can potentially reveal some personality aspects of its author.

### Reflection: personality projection in the self-portrait

Personality related cues can be retrieved from a person's photographs, objects and behavior and provide a solid basis for personality judgments by unfamiliar others (overview in Qiu et al., 2015). In this regard, the process of personality assessment is similar to the basic logic of projective techniques. Those techniques are based on observation and interpretation of a person's responses to stimulation of the imaginative processes (Murray, 1938).

From this perspective, a selfie could be compared to constructive tests in the scope of projective techniques. In constructive tests, the respondent creates some previously non-existent object in response to a few very broad directions (Bornstein, 2007). A selfie is a construction used by an author to explore and share his/her own identity. This is similar to a respondent in a Draw-a-Person (DAP) test (Machover, 1951), who constructs a drawing that represents the author himself (Craddick, 1963). In projective drawing, psychomotor activity is captured on paper (Hammer, 1968), and in the case of a selfie, the same is captured in a snapshot. The content of the output is determined by conscious and unconscious perceptions of the self and the environment (Hammer, 1968).

One stimulus that initiates construction of the selfie is one's intention to present oneself before some audience. Hence, the author of the selfie intentionally expresses him or herself in such a way as to achieve a certain impression. However, besides the intentionally given expression, the same act also “gives off” an expression that is unintentionally revealed (Goffman, 1959). In the interaction at hand, such expressions play an important role in impression management and contribute to our understanding of the messages received. Behind this interplay of expressions, we assume that personality traits will provide a structure for what is projected.

### The present studies

Based on the previous research, the phenomenon of the selfie can be explored meaningfully from three perspectives: the selfie as picture, the selfie as reflection and the selfie as impression. From the first perspective, the selfie can be analyzed in the context of (self-portrait) photography, with the main focus on the visual elements or cues in the picture, their position and relations; from the second perspective, distinct cues in the selfie picture can be related to the personality characteristics of the author of the picture; and from the last perspective, the selfie is interpreted in the context of the impression formation created in others by the selfie picture.

In this context, we invited students to participate in a series of psychological studies exploring personality concepts, self-presentation and information technology use and focused our investigation on the first two perspectives of selfie exploration.

In the first part of the investigation (Study 1), the concept of the selfie was defined to students, and they were asked to each submit a freely chosen selfie with information about its availability to others (from private to completely available). With this strategy, we allowed students to reflect before deciding which selfie they should send; thus, we potentially fostered a more active relation between the author of the selfie and the product (the selfie picture).

The research focus of Study 1 was on the first perspective—i.e., the selfie as picture, aimed at answering the question of whether it is possible to analyze selfies systematically to build a valid and reliable coding scheme. According to the resulting coding categories, we further analyzed selfies in relation to gender and degree of availability to others.

After a delay (8–12 weeks), the same groups of students were asked to participate in the second part of the investigation (Study 2). They were asked to complete the survey battery, comprising a range of personality concepts and concepts related to information technology use. The special focus of Study 2 is thus exploration of potential relations between the coding of the selfies (according to the coding scheme from Study 1) and selected personality concepts (see Measures in Study 2) and indicators of information technology use.

Our approach to analysing selfies was similar to those used in previous research (e.g., Qiu et al., 2015; Eager and Dann, 2016). In line with Qiu et al. (2015), we used a more elaborate coding scheme and more personality constructs. We focused more on the visual cues of selfie pictures (i.e., what is shown) and on the relations of these cues to the personality characteristics of their authors and not on the impression created by the selfie, as was the case in a study by Eager and Dann (2016), which focused on what is seen and the story behind it.

Next, our research deliberately refrained from discussing the validity of projective techniques, which have been extensively criticized, especially in the US, while still attracting scholarly interest and value in clinical settings (Piotrowski, 2015a,b). This study aims to check personality projection into a selfie by using established psychological instruments.

Based on the literature review and the relative lack of comparative studies, we formulated some initial hypotheses:

In the context of Study 1, we expected that, according to the study of Qiu et al. (2015):

*H1: Selfie pictures could be objectively and reliably decomposed into distinct visual cues and that a subsequent coding scheme could be elaborated*.

In the context of the selfie as photography and, according to Tifferet and Vilnai-Yavetz (2014), we presupposed:

*H2: More women would have selfies exhibiting eye contact and positive mood*,

*H3: Men would have more selfies made in the public sphere*.

Studies by Bruno et al. (see Bruno and Bertamini, 2013: Bruno et al., 2015) showed that there exists an unconscious, culture-independent preference for displaying one's left cheek. This results in a left-cheek bias in the case of standard selfies, whereas mirror-style selfies have right-cheek bias. According to Bruno, this effect originates from lateral asymmetries in processing faces. We further presupposed that, in the majority of standard -style selfies:

*H4: The left side of the face in standard-style selfies would be emphasized*.

In accordance with Döring et al. (2016), we expected:

*H5: Women in selfies would more often tilt their heads or bodies*.

Initial studies of face-ism (e.g., Archer et al., 1983; Szillis and Stahlberg, 2007) indicated that women would have pictures with a smaller proportion of the face to the total picture. However, contrary to the above-mentioned assumption of the sexual objectification of women, selfies as self-portraits are also a potential means for women's emancipation (e.g., Warfield, 2014) and consequently we expected:

*H6: There would be no gender differences in selfies regarding face-ism*.

Relating characteristics of selfie pictures to psychological concepts and concept of NPI (Narcissistic Personality Inventory), in accordance with Giessner et al. (2011), we expected:

*H7: Selfies with lower camera position would be related to higher scores on the authority factor*.

A similar trend can be expected for the concept of masculinity or, in the opposite direction, for femininity:

*H8: Selfies with lower camera position are related to higher scores on masculinity and lower scores on femininity*.

Finally, all assumptions about camera position can be attributed to the head position in the selfie pictures:

*H9: Selfies with the head in the upper regions of the pictures are related to higher scores on authority and masculinity and lower scores on femininity*.

## Study 1

### Method

#### Participants

Initially, 234 students were invited to participate in the investigation. They were recruited from a range of fields of study [psychology (30.3%), sociology (14.5%), pedagogy (14.6%), architecture (19.7%) and civil engineering (20.9%)] from the public university in Slovenia (University of Maribor). There were 73.5% females, and the mean age for the total sample was 20.34 years (*SD* = 1.43).

One hundred and sixty-five students from the initial groups of students sent selfies and thus actively participated in the study (70.5% response rate from the initial group). There were 76.4% females, and the mean age in the sample was 20.30 years (*SD* = 1.4). The participants received no financial compensation for their involvement in the study.

#### Procedure

The researcher explained the concept of the selfie to the target groups of students, and they were invited to participate in the study by submitting one freely chosen selfie picture. With this strategy, students were allowed to reflect before choosing which selfie they should send; thus, we potentially fostered a more active relation between the author of the selfie and the product (the selfie picture). Participants were also asked to provide information about the availability of the selfie, i.e., who could have access to the selfie, and received an individual code to provide for anonymity of the participants in later data processing phases. Selfie availability was later divided into two groups: an intimate circle (people close to the author) and a social network (available to everyone using SNS). Independent raters coded each selfie picture according to the coding scheme developed during the study (detailed description in the next section). Ethical review and approval was not required for this study in accordance with the national and institutional guidelines.

We tested H1 with measures of interrater reliability, Fleiss' kappas, and we used Chi square statistics for analysing H2, H3, H4, and H5. We ran each analysis separately. We tested H6 using the *t*-test. For analysing context and head position, we used the Mann-Whitney test.

#### Coding

The coding of the selfies followed the general principles of grounded theory (Glaser and Strauss, 1967). In the initial brainstorming phase, the researchers composed a set of categories for coding the selfies. In the second phase, three raters individually coded a small set of selfies (testing sample) and discussed each coded selfie collectively. The result of this phase was the elaborated scheme for coding the selfies.

In the subsequent phase, three independent raters individually coded all selfies received. In the process of coding, any potential new category for coding was discussed collectively and, with the consent of raters, added to the coding scheme and to the re-coding of already coded selfies. In the next phase, data from all raters were collected, intererater reliabilities were calculated (Fleiss' kappas) and selfies with less than 2/3 agreement were collectively discussed to achieve consensus among the raters. The coding scheme from this phase includes the following categories:

##### Background brightness

We used dichotomous coding (1-light; 2-dark) to determine the brightness of each photo.

##### Context

We identified seven codes for the different contexts in which participants took their selfies. Codes were 1 (room), 2 (free time), 3 (outside), 4 (car), 5 (recreation—sportslike activities), 6 (bathroom), and 7 (public transportation). Some selfies could be placed under more than one code, on account of featuring multiple contexts. In these cases, we picked the category that stood out the most and that had been chosen by the majority of the raters. Subsequently, we merged the seven categories into two: 1 (inside) and 2 (outside).

##### Tilt of the body

We coded the position of the body with the help of diagonals. Left diagonal (LD) reached from the upper left corner of the photo to the right bottom corner. Right diagonal (RD) reached from the upper right corner to the left bottom corner of the photo. We coded as follows: Center (C), body not inclined to either side; LD, body leaning in accordance with the left diagonal; RD, body leaning in accordance with the right diagonal.

##### Tilt of the head

As with the previous category, we coded the position of the head but we didn't use diagonals. If the head was not inclined, we coded as center (C). If the head was tilted to the left so that the left ear was approaching the left shoulder, we coded as a left tilt (L); the same criterion went for a right tilt (R). If the head was bent forward and the chin directed toward the chest, we coded forward (F), and if the head was tilted back, we coded back (B). Rotation of the head either to the left or right side was coded as “C” only if the head wasn't tilted. If the head was tilted, we coded as described here.

##### Part of the face

We coded the side of the face (L or R) which was in the spotlight and more visible to the viewer. If neither side was prevalent, we coded center (C).

##### Eye contact

If the gaze was directed to the person looking at the photo, we coded this as eye contact. Additionally, we coded if the person wore glasses, sun glasses, ski goggles or something else. For further analysis, we used dichotomous coding: 1 (eye contact) and 2 (no eye contact).

##### Frame of the picture

We coded the orientation of picture frame. These codes were horizontal, vertical and square.

##### Head position

We divided the photo with horizontal and vertical line and then determined the position of the head. These codes are as follows: Left up and down (LU and LD); right up and down (RU and RD), center up, down, and center (CU, CD, and C) and center left and right (CL and CR).

##### Mood

We coded three different expressions: positive, negative and neutral. By positive expression, we mean a smile or expressions resembling a smile (a positive mood). The category negative was assigned to expressions expressive of sadness, disgust etc. The neutral category included all other expressions that weren't explicitly positive or negative, a “serious face.”

##### Social distance

According to Hall and Pennington (2013; see also Kress and van Leeuwen, 2006), we coded six social distances. At intimate distance (INT) we see the face or head only. At close personal (CP) distance, we see the head and shoulders. At far personal distance (FP), we see the person from the waist up; at a close social distance (CSD), we see the whole figure. At far social distance (FSD), we see the whole figure with the space around it, and at public distance (PD), we can see the torsos of at least four or five people.

##### Camera position

This represents the camera position from which the selfie is taken. The codes are as follows: Right side up, center and down (RU, RC, and RD); left side up, center and down (LU, LC, and LD) and central (front) position up, center and down (FU, FC, and FD).

##### Face-ism

From the concept of face-ism (Archer et al., 1983), a ratio was calculated of (a) the distance from the top of the head to the lowest point of the chin, and (b) the distance from the top of the head to the lowest visible part of the body in the photo. When the body axis of the person depicted in the photo was tilted, prior to measurement the photo was rotated. The face-ism index was measured with Fiji software (Schindelin et al., 2012).

##### Other

This category reflects observations of the particularities of the selfies that could not be classified by any of the previously mentioned coding categories [e.g., specific expression, pose, touching hair; number of other persons in group selfies (groupies)]. Special cases involved effects that participants used to alter the selfie or their self-presentation (e.g., black/white, color filter).

**Figure 1:**
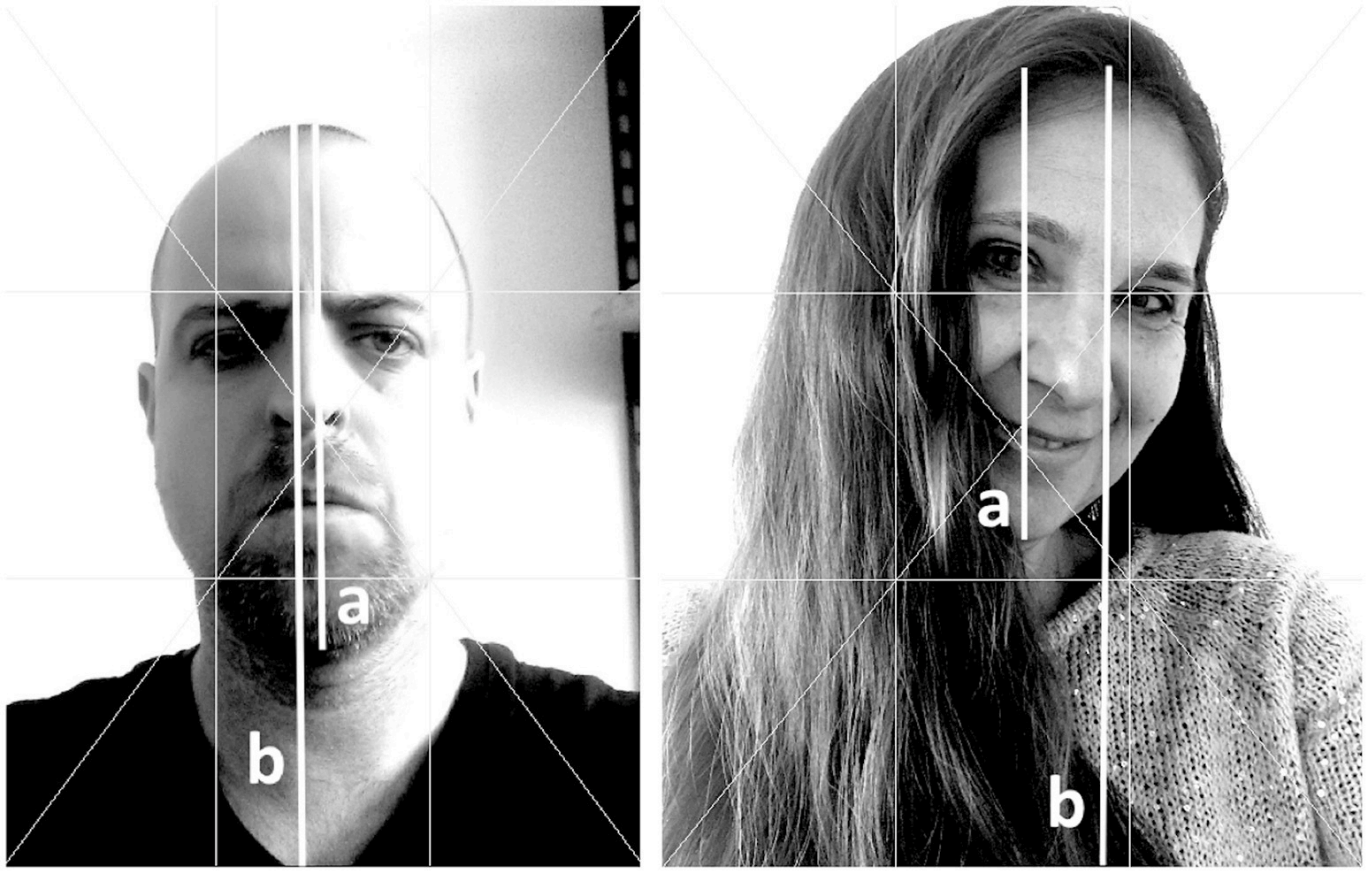
**Examples of male and female selfies in Fiji, with horizontal, vertical and diagonal lines indicated and additional lines for the face-ism calculation (a/b)**.

In a subsequent phase, coding categories with low interrater reliability (*Tilt of the body, Tilt of the head, Head position*, and *Camera position*) were coded once again by two independent raters in the image processing package Fiji, with horizontal, vertical and diagonal lines indicated on the selfie pictures. Cohen's kappas for two raters were calculated. An example of a selfie picture is shown in Figure 1.

### Results and discussion

Table 1 shows the results of the selfie coding, i.e., main categories with frequencies and percentages for each sub-category of the coding scheme, with the most frequent sub-categories in bold. For each category of coding the result of interrater reliability (Fleiss' kappa) is in the last column.

**Table 1:** **Selfie coding categories and subcategories**.

**Main category**	**Sub-category**	**Frequency**	**Percentage**	**Kappa^a^**
Background brightness	Light	**137**	**83**	0.68
	Dark	27	16.4	
Context	Room	**98**	**59.4**	0.82
	Free time	19	11.5	
	Outside	16	9.7	
	Car	15	9.1	
	Recreation	10	6.1	
	Bathroom	5	3.0	
	Public transport	1	0.6	
Tilt of the body	LD	29	17.6	0.88 (0.25)^c^
	RD	37	22.4	
	C	**99**	**60**	
Tilt of the head	L	12	7.3	0.67 (0.50)^c^
	R	23	13.9	
	C	**130**	**78.8**	
Part of the face	L	38	23	0.66
	R	52	31.5	
	C	**75**	**45.5**	
Eye contact	Yes	**105**	**63.6**	0.88
	No	29	17.6	
	Glasses	13	7.9	
	Sun glasses	2	1.2	
	Ski goggles	16	9.7	
Frame of the picture	Horizontal	40	24.2	0.91
	Vertical	**99**	**60**	
	Square	26	15.8	
Head position	LU	4	2.4	0.97 (0.32)^c^
	LD	2	1.2	
	RU	1	0.6	
	RD	2	1.2	
	CU	57	34.5	
	CD	1	0.6	
	CL	21	12.7	
	CR	8	4.8	
	C	**69**	**41.8**	
Mood	Positive	**121**	**73.3**	0.60
	Negative	–	–	
	Neutral	44	26.7	
Social distance	INT	2	1.2	0.82
	CP	**120**	**72.7**	
	PD	5	3.0	
	CSD	1	0.6	
	FSD	19	11.5	
	FP	18	10.9	
Camera position	RU	3	1.8	0.23 (0.30)^c^
	RC	11	6.7	
	RD	17	10.3	
	LU	15	9.1	
	LC	35	21.2	
	LD	**40**	**24.2**	
	CU	8	4.8	
	CC	24	14.5	
	CD	12	7.3	
Face-ism index^b^		0.55	0.12	

*Bold numbers represent modal values in each category*.

a *Fleiss' kappa*.

b *Values in the columns are mean and standard deviation*.

c *Cohen's kappa values of two raters using Fiji and initial Fleiss' kappa values in brackets (first coding)*.

According to the modal values of sub-categories, the modal selfie from our sample is in a vertical frame, with a light background, taken in an inside context (a room). Generally, body and head are not tilted, and the face is centrally exhibited (C). The head is in the central (C) to central upper (CU) position in the picture, and the camera is in the left down (LD) or left center (LC) position. The actors in the selfies are at a close personal distance (CP), with eye contact and mostly exhibiting a positive mood.

On average, the measures of intererater reliability, Fleiss' kappas, show good agreement between raters (see Fleiss et al., 2003). However, there are differences between categories. *Frame of the picture, eye contact, context*, and *social distance* are categories with high kappa values, consequently reflecting a high level of agreement among raters; while the *tilt of the body, camera position*, and *head position* categories have relatively low kappa values from the first phase of coding and reflect less agreement between raters. In the subsequent phase, using Fiji, the kappas of coding categories that initially had relatively low kappas and the use of additional lines to improve the coding, substantively improve, with the exception of *camera position*, which we excluded from all subsequent analyses.

In the additional category *Other*, there were particular attributes or qualities of the selfies that could not be classified by the existing coding categories.

In our sample (*N* = 165) one selfie (0.6%) was taken in a mirror; one participant (0.6%) took a selfie with an animal; 1.8% (*f* = 3) of participants wore a mask in their selfie; 2.4% (*f* = 4) of participants wore a helmet; 4.8% (*f* = 8) of participants touched their hair while taking the selfie. 3% (*f* = 5) of participants sent us a group selfie (groupies), which means that the selfie included 3 or more people. 3.6% (*f* = 6) of the selfies in the study were not taken by a participant in the study. Despite the criterion in the definition that a selfie must be taken by the subject (actor) in the picture, we decided not to exclude these selfies from the study. They still represented the perception of study participants about what a selfie means to them and were thus representations of themselves, even though not meeting the technical criterion of a selfie.

A special case in the category *Other* were those selfies where the participants (actors) used effects to alter the appearance of the selfie. Some selfies were probably taken by mobile phones that have effects built into their default camera applications and these effects discreetly enhance the selfie. In this group of visibly altered selfies (19.4%, *f* = 32) there were three sub-groups. Most participants (7.3%, *f* = 12) altered their selfie by adding a black and white effect; 6.7% (*f* = 11) of participants altered their selfie by adding a portrait effect; some participants (5.5%, *f* = 9) altered their selfie by adding a color effect. Because of the heterogeneity of this category, we didn't include it in the coding scheme, and preliminary statistical analysis didn't indicate any significant relation to other coding categories or other constructs in Study 2. Additionally, there were no indices of selfie stick use, and four participants made mirror-style selfies with all actors in the pictures in a central position for the body, head and part of the face.

#### Selfie by gender and context

In the following section we analyze selfie pictures by gender of the actor and context in the selfie using the Chi square and Mann-Whitney test in IBM SPSS 23. Both these factors can be interpreted as contextualized or input variables, i.e., who took the selfie and in what kind of context the selfie was taken.

For further analysis, categories from the initial coding scheme in Table 1, *tilt of the head, context* and *social distance*, were dichotomized (center vs. tilted for *tilt of the head*, inside vs. outside for *context*, personal vs. social for *social distance*); and the initial category *head position* was transformed into dimensional categories (*head position – abcissa, head position – ordinate*).

More detailed analysis of the sub-categories of the coding scheme indicates that male and female selfies differ, especially in the categories *tilt of the head, part of the face, head position*, and *mood*.

The statistical analysis, yielded a significant association between gender and head tilting $\chi_{\left(1\right)}^{2}$ = 13.75, *p* < 0.001. A moderate association emerged between males with their heads in the center and females tilting their heads Cramer's V = 0.28, *p* < 0.001. None of the male participants tilted their heads, while more than half the female participants tilted their heads either to the right or to the left.

We found a difference in gender and the representation of the side of the face. Males preferred looking straight at the camera, resulting in a more central position of the face than for females [$\chi_{\left(2\right)}^{2}$ = 9.3, *p* < 0.01]. Women preferred either the right side (32.5%) or the left side (27.8%) of the face.

Head position in males differed significantly from that in females *U* = 1835, *p* < 0.01, *r* = −0.21. Male heads were positioned in the center of the selfie, while females positioned their heads in the upper region of the selfie.

In the category of mood, we found a significant association between men and neutral expressions and an association between women and positive mood [$\chi_{\left(1\right)}^{2}$ = 15.82, *p* < 0.001]. There is a moderate association between men and neutral expressions and women and positive expressions (Cramer's *V* = 0.31, *p* < 0.001).

In sum, in contrast to the typical female selfie, the head of the actor in the male selfie is not tilted; the face is more centrally located and placed in the center of the picture, with the actor exhibiting neutral to positive mood. In Figure 1, the left-hand picture represents a typical male selfie and the right-hand picture, a typical female selfie.

Analysis of the context of the pictures revealed only that head position differed statistically according to context *U* = 1994, *p* < 0.01, *r* = −0.23. When in an outside context (*Mdn* = 1), the participant's head was in the lower part of the selfie, while for an inside context (*Mdn* = 2), the head was in the upper part of the selfie.

However, in accordance with our initial assumption, there were no significant differences between male (*M* = 0.53, *SE* = 0.01) and female (*M* = 0.55, *SE* = 0.01) selfies in face-ism *t*_(73)_ = −0.69, *p* = 0.48.

## Study 2

### Method

#### Participants

128 students (78.1% females) who had initially sent us selfie pictures (Study 1) participated in the second part of the study (77.6% response rate from Study 1). The mean age for this sample was 20.30 years (*SD* = 1.41); participants ranged from 19 to 28 years old.

#### Procedure

The participants were asked to complete the survey battery, comprising a range of personality concepts and concepts related to information technology use. They marked the instrument with the same individual code they had received in the previous study. Data from both studies were merged according to the codes by an independent researcher who did not participate in the data gathering phases of either study.

In statistical analysis, we used *t*-tests, but we didn't analyse H7 and H8, owing to low interrater reliability of the category camera position. Independent *t*-tests were performed, with groups according to coding categories and variation of psychological constructs in each test. We also corrected the significance level according to the number of analyses which included the same grouping variable (i.e., Bonferroni correction). For analysing H9, we used Spearman's rho correlation coefficient. Pearson correlation was used to determine possible connections between psychological constructs and the face-ism index.

#### Measures

The complete survey battery in the second part of the investigation comprised of the Narcissistic Personality Inventory (NPI; Raskin and Terry, 1988), part of the Self-Description Questionnaire III (SDQ-III; Marsh and O'Neill, 1984), the Bem Sex-Role Inventory (BSRI; Bem, 1974), the Big Five Inventory (BFI; John and Srivastava, 1999) and the Facebook Intensity Scale (FBI; Ellison et al., 2007). For the survey battery, we used the all the questionnaires, as described below, except for SDQ III. For the purposes of our study, we used only the subscale “physical appearance” from SDQ III, which comprises 10 items.

*NPI* (Raskin and Terry, 1988) is a 40-item self-report questionnaire for assessing narcissism as a personality characteristic. Each item consists of a pair of narcissistic and non-narcissistic statements, but for the purpose of our study, we measured statements on a 5-point rating scale (1 = strongly disagree; 5 = strongly agree). According to the authors of the NPI (Raskin and Terry, 1988), the questionnaire consists of 7 dimensions. Cronbach alphas for our sample were as follows: authority (8 items; α = 0.80), exhibitionism (7 items; α = 0.62), superiority (5 items; α = 0.56), entitlement (6 items; α = 0.60), exploitativeness (5 items; α = 0.62), self-sufficiency (6 items; α = 0.46) and vanity (3 items; α = 0.72). Unlike Raskin and Terry (1988), we experienced some difficulties with the NPI structure in our sample, like many other authors (Emmons, 1987; Kubarych et al., 2004; Ackerman et al., 2011). Reviewing research findings by other authors (Emmons, 1987; Kubarych et al., 2004; Corry et al., 2008; Ackerman et al., 2011), one finds that the dimensions of authority and exhibitionism are the most frequently reoccurring ones. As in the study by Raskin and Terry (1988) authority and exhibitionism have the biggest positive correlation value and one of the highest internal consistency score among all dimensions. Because of the greater comparability of our findings to other (potential) studies, we included all the original NPI dimensions, but focused special attention on the dimensions of authority and exhibitionism in subsequent analyses.

*BFI* (John and Srivastava, 1999) is a 44-item questionnaire measuring five personality traits. All items are measured on a 5-point rating scale (1 = strongly disagree; 5 = strongly agree). Reliability for each dimension in our sample was as follows: extraversion (8 items; Cronbach's α = 0.82), openness (10 items; α = 0.83), conscientiousness (9 items; α = 0.77), agreeableness (9 items; α = 0.72) and for neuroticism (8 items; α = 0.79).

*BSRI* (Bem, 1974) is a short version, which consists of 30 personality characteristics. For the purpose of our study we used a 5-point Likert scale ranging from 1 (never or almost never true) to 5 (always or almost always true). Ten of the characteristics are stereotypically feminine, ten are stereotypically masculine, and ten are considered neutral. Reliability for our sample for feminine items is (α = 0.82), for masculine (α = 0.76) and for neutral (α = 0.40). In all our analyses we used subscales of femininity and masculinity.

*SDQ III* (Marsh and O'Neill, 1984) is a self-report questionnaire designed to measure 13 factors of self-concept. For the current research purposes, we used 10 items on a 5-point rating scale, ranging from 1 (definitely false) to 5 (definitely true). Cronbach alpha for physical appearance in our sample was 0.87.

*FBI* (Ellison et al., 2007) is used to measure Facebook use. The first six items are measured on a 5-point Likert scale ranging from 1 (strongly disagree) to 5 (strongly agree). The seventh and eighth items are self-report, open-ended questions about the number of friends and amount of time spent on Facebook. Following the recommendations of the FBI authors, we transformed open-ended responses into five approximately equal groups, from low to high intensity users (with respect to number of friends and time spent on Facebook). Cronbach alpha for our sample was 0.84.

### Results and discussion

In Table 2 are the descriptive statistics for psychological constructs (NPI, BFI, BSRI, SDQ - physical appearance) and related concepts (FBI), according to the sub-categories from the coding scheme of selfies. From the initial coding scheme (Study 1) we used dichotomized categories for *tilt of the head, context* and *social distance*; and for the category *head position*, dimension categories (*head position – abcissa, head position – ordinate*).

**Table 2:** **Descriptive statistics of selfie coding categories regarding psychological constructs and FBI**.

**Category**	**Sub category^a^**		**BFI**					**NPI**							**SDQ**	**BSRI**		**FBI**
			**E**	**A**	**C**	**N**	**O**	**Au**	**Ex**	**Su**	**En**	**Exp**	**Ss**	**Va**		**M**	**F**
Background	Light		3.64	3.71	3.77	2.85	3.80	3.02	2.23	2.90	3.12	2.86	3.18	2.39	3.44	3.39	4.00	2.90
brightness	(107)		(0.69)	(0.58)	(0.55)	(0.61)	(0.61)	(0.66)	(0.55)	(0.58)	(0.60)	(0.58)	(0.50)	(0.78)	(0.61)	(0.52)	(0.55)	(0.76)
	Dark		3.42	3.35	3.53	3.02	3.76	2.95	2.28	2.54	3.24	2.96	3.03	2.09	3.02	3.39	3.86	3.01
	(21)		(0.52)	(0.65)	(0.54)	(0.56)	(0.45)	(0.51)	(0.45)	(0.54)	(0.59)	(0.45)	(0.43)	(0.69)	(0.51)	(0.33)	(0.45)	(0.75)
	Inside		3.60	3.61	3.71	2.91	3.76	3.00	2.24	2.86	3.18	2.92	3.15	2.30	3.34	3.38	3.97	3.01
Context	(89)		(0.69)	(0.62)	(0.56)	(0.63)	(0.60)	(0.64)	(0.54)	(0.59)	(0.61)	(0.54)	(0.52)	(0.76)	(0.61)	(0.50)	(0.57)	(0.73)
	Outside		3.59	3.75	3.79	2.80	3.85	3.02	2.23	2.80	3.05	2.78	3.16	2.44	3.43	3.42	4.01	2.78
	(39)		(0.64)	(0.56)	(0.53)	(0.54)	(0.55)	(0.63)	(0.53)	(0.59)	(0.57)	(0.59)	(0.42)	(0.78)	(0.62)	(0.47)	(0.48)	(0.67)
	LD		3.90	3.73	3.82	2.78	3.99	3.17	2.19	2.89	3.06	2.93	3.13	2.46	3.63	3.55	4.13	2.84
Tilt of the body	(23)		(0.53)	(0.63)	(0.41)	(0.63)	(0.60)	(0.56)	(0.56)	(0.70)	(0.54)	(0.52)	(0.32)	(0.93)	(0.58)	(0.35)	(0.42)	(0.89)
	RD		3.52	3.65	3.67	2.79	3.61	2.87	2.14	2.69	3.18	2.83	3.17	2.02	3.22	3.33	3.94	3.05
	(29)		(0.76)	(0.68)	(0.47)	(0.62)	(0.65)	(0.71)	(0.41)	(0.53)	(0.61)	(0.65)	(0.62)	(0.67)	(0.59)	(0.56)	(0.57)	(0.50)
	C		3.54	3.63	3.73	2.94	3.8	3.01	2.29	2.88	3.15	2.88	3.16	2.43	3.35	3.36	3.95	2.89
	(76)		(0.66)	(0.60)	(0.61)	(0.59)	(0.54)	(0.62)	(0.57)	(0.57)	(0.62)	(0.54)	(0.48)	(0.72)	(0.62)	(0.56)	(0.56)	(0.79)
	Cente		3.58	3.67	3.72	2.88	3.87	3.06	2.23	2.81	3.16	2.87	3.19	2.32	3.37	3.41	3.97	2.88
Tilt of the head	(98)		(0.71)	(0.58)	(0.55)	(0.64)	(0.56)	(0.67)	(0.51)	(0.59)	(0.61)	(0.58)	(0.48)	(0.74)	(0.63)	(0.50)	(0.49)	(0.76)
	Tilted		3.69	3.61	3.77	2.86	3.54	2.82	2.26	2.93	3.08	2.89	3.05	2.41	3.37	3.34	4.01	3.02
	(30)		(0.53)	(0.68)	(0.54)	(0.49)	(0.60)	(0.49)	(0.62)	(0.59)	(0.58)	(0.47)	(0.52)	(0.85)	(0.57)	(0.47)	(0.68)	(0.70)
	Left		3.47	3.75	3.84	2.97	3.92	2.88	2.19	2.88	3.05	2.89	3.06	2.17	3.20	3.29	4.09	2.75
Part of the face	(30)		(0.71)	(0.49)	(0.55)	(0.55)	(0.65)	(0.64)	(0.62)	(0.70)	(0.59)	(0.61)	(0.49)	(0.75)	(0.55)	(0.44)	(0.52)	(0.73)
	Right		3.66	3.60	3.74	2.75	3.72	3.02	2.25	2.86	3.22	2.87	3.23	2.26	3.36	3.44	3.93	3.09
	(39)		(0.63)	(0.67)	(0.54)	(0.62)	(0.50)	(0.64)	(0.48)	(0.52)	(0.68)	(0.53)	(0.48)	(0.70)	(0.58)	(0.43)	(0.54)	(0.71)
	Center		3.63	3.64	3.68	2.92	3.77	3.06	2.25	2.80	3.13	2.87	3.15	2.49	3.46	3.41	3.96	2.94
	(59)		(0.68)	(0.62)	(0.56)	(0.62)	(0.60)	(0.63)	(0.54)	(0.58)	(0.55)	(0.56)	(0.49)	(0.80)	(0.65)	(0.55)	(0.55)	(0.71)
	Yes		3.57	3.61	3.73	2.93	3.74	3.02	2.26	2.82	3.20	2.93	3.15	2.33	3.32	3.41	3.94	2.97
Eye contact	(83)		(0.64)	(0.61)	(0.52)	(0.59)	(0.57)	(0.61)	(0.56)	(0.59)	(0.58)	(0.54)	(0.49)	(0.73)	(0.62)	(0.46)	(0.57)	(0.72)
	No		3.50	3.73	3.81	2.87	3.80	2.85	2.04	2.87	2.92	2.75	3.21	2.28	3.49	3.23	4.13	2.87
	(23)		(0.81)	(0.62)	(0.53)	(0.65)	(0.72)	(0.76)	(0.31)	(0.5)	(0.48)	(0.62)	(0.48)	(0.74)	(0.62)	(0.61)	(0.41)	(0.81)
	Horizontal		3.80	3.86	3.86	2.65	3.92	3.07	2.12	2.85	3.09	2.85	3.24	2.38	3.43	3.38	4.15	2.78
Frame of the picture	(31)		(0.53)	(0.50)	(0.56)	(0.55)	(0.62)	(0.66)	(0.45)	(0.48)	(0.60)	(0.57)	(0.48)	(0.72)	(0.54)	(0.47)	(0.48)	(0.75)
	Vertical		3.56	3.57	3.7	2.92	3.78	3.04	2.28	2.81	3.22	2.97	3.16	2.32	3.36	3.44	3.88	2.88
	(77)		(0.67)	(0.61)	(0.55)	(0.63)	(0.58)	(0.59)	(0.53)	(0.60)	(0.58)	(0.51)	(0.49)	(0.69)	(0.64)	(0.43)	(0.52)	(0.77)
	Square		3.43	3.65	3.65	3.08	3.65	2.78	2.25	2.94	2.89	2.56	2.99	2.40	3.31	3.22	4.09	3.25
	(20)		(0.84)	(0.68)	(0.53)	(0.51)	(0.53)	(0.75)	(0.66)	(0.69)	(0.62)	(0.61)	(0.47)	(1.09)	(0.64)	(0.70)	(0.65)	(0.62)
		Down	3.55	3.95	3.93	2.65	3.56	3.12	2.00	2.52	3.00	2.80	3.13	2.06	3.38	3.38	4.16	2.67
Head position	Ordinate	(5)	(0.85)	(0.51)	(0.70)	(0.56)	(0.49)	(0.71)	(0.34)	(0.64)	(0.78)	(0.20)	(0.44)	(0.76)	(0.84)	(0.52)	(0.28)	(0.64)
		Center	3.55	3.68	3.75	2.83	3.80	3.02	2.26	2.88	3.08	2.88	3.19	2.36	3.42	3.39	3.94	2.87
		(76)	(0.68)	(0.63)	(0.51)	(0.64)	(0.53)	(0.62)	(0.58)	(0.56)	(0.57)	(0.53)	(0.49)	(0.81)	(0.60)	(0.47)	(0.58)	(0.79)
		Up	3.70	3.58	3.68	2.98	3.80	2.96	2.23	2.79	3.25	2.87	3.09	2.35	3.27	3.38	4.02	3.02
		(47)	(0.64)	(0.58)	(0.59)	(0.55)	(0.68)	(0.67)	(0.48)	(0.62)	(0.61)	(0.64)	(0.50)	(0.71)	(0.61)	(0.52)	(0.49)	(0.69)
	Abscissa	Left	3.47	3.63	3.77	3.00	3.73	2.92	2.16	2.79	2.99	2.77	3.10	2.11	3.27	3.32	4.02	2.92
		(20)	(0.91)	(0.68)	(0.57)	(0.63)	(0.68)	(0.74)	(0.42)	(0.53)	(0.59)	(0.70)	(0.55)	(0.81)	(0.71)	(0.54)	(0.51)	(0.54)
		Center	3.63	3.65	3.71	2.87	3.82	2.99	2.25	2.85	3.19	2.91	3.15	2.35	3.37	3.39	3.98	2.94
		(98)	(0.62)	(0.58)	(0.54)	(0.60)	(0.56)	(0.62)	(0.55)	(0.60)	(0.60)	(0.54)	(0.49)	(0.75)	(0.59)	(0.49)	(0.53)	(0.77)
		Right	3.57	3.71	3.85	2.71	3.69	3.33	2.28	2.80	2.98	2.78	3.3	2.76	3.51	3.53	3.95	2.7
		(10)	(0.63)	(0.77)	(0.64)	(0.62)	(0.66)	(0.48)	(0.64)	(0.66)	(0.59)	(0.44)	(0.34)	(0.78)	(0.66)	(0.37)	(0.70)	(1.00)
Mood	Positive		3.60	3.62	3.74	2.92	3.80	2.98	2.26	2.87	3.17	2.86	3.14	2.40	3.41	3.40	4.02	2.98
	(95)		(0.68)	(0.61)	(0.53)	(0.58)	(0.57)	(0.65)	(0.55)	(0.58)	(0.59)	(0.54)	(0.50)	(0.80)	(0.60)	(0.51)	(0.55)	(0.70)
	Neutral		3.6	3.77	3.70	2.75	3.78	3.07	2.15	2.73	3.06	2.92	3.19	2.19	3.25	3.36	3.85	2.81
	(33)		(0.66)	(0.60)	(0.62)	(0.67)	(0.63)	(0.59)	(0.49)	(0.63)	(0.64)	(0.61)	(0.44)	(0.65)	(0.66)	(0.44)	(0.50)	(0.78)
Social distance	Personal		3.61	3.65	3.72	2.88	3.81	2.99	2.25	2.86	3.15	2.91	3.14	2.37	3.36	3.38	3.98	2.94
	(107)		(0.69)	(0.61)	(0.54)	(0.63)	(0.58)	(0.63)	(0.55)	(0.61)	(0.59)	(0.55)	(0.48)	(0.80)	(0.62)	(0.50)	(0.55)	(0.75)
	Social		3.58	3.67	3.79	2.87	3.70	3.08	2.15	2.73	3.10	2.70	3.20	2.23	3.41	3.41	3.99	2.93
	(21)		(0.60)	(0.58)	(0.62)	(0.45)	(0.60)	(0.67)	(0.48)	(0.48)	(0.66)	(0.57)	(0.52)	(0.55)	(0.61)	(0.47)	(0.52)	(0.59)
Face-ism			−0.20	0.03	0.13	0.14	0.03	−0.12	0.00	−0.01	−0.27^*^	−0.11	−.010	−0.09	−0.07	−.019	0.07	−0.08
index^b^																		

*BFI factors: extraversion (E), agreeableness (A), conscientiousness (C), neuroticism (N) and openness (O). NPI dimensions: authority (Au), exhibitionism (Ex), superiority (Su), entitlement (En), exploitativeness (Exp), self-sufficiency (Ss) and vanity (Va). BSRI subscales: masculinity (M), femininity (F). Values are represented with mean and standard deviation in brackets. Bold numbers represent statistically significant results*.

a *Numerus in brackets for each subcategory*.

b Pearson's correlation with psychological constructs (in the same order as noted);

* *p < 0.05*.

We statistically analyzed relations between the selfie coding categories, psychological constructs and FBI using the *t*-test and Pearson's correlation. Generally, the analyses yielded statistically significants results, which are identified in bold text in Table 2.

There are no significant relations between coding cues and psychological constructs.

Entitlement is the only construct (from NPI) that had a significant correlation with the face-ism index (*r* = −0.27).

Additionally, we analyzed the relation between all the concepts and the availability of selfie pictures to others. The results for participants whose selfie was available on social networks (*M* = 3.07, *SE* = 0.079) showed more emotional stability [*t*_(124)_ = −2.93, *p* < 0.01] than those whose selfie was available only to an intimate circle of people (*M* = 2.75, *SE* = 0.070), with an effect size of *r* = 0.27.

## General discussion

From the perspective of the average user/observer of social networks, the interpretation and consequent meaning of a selfie is related to limited capacity to process and understand the context of the selfie picture. Computer based algorithms have the advantage of efficiently processing large amounts of (meta)data in profiling individual users of social networks. However, the incidental user can creatively focus attention on particular attributes (cues) of the selfie and the context of the picture and over time gradually elaborate an impression of the actor in the selfie.

Qiu et al. (2015) emphasize the lens model as a useful framework in the process of assessment of personality characteristics on the basis of the selfie picture. According to the lens model, in the interpretation of a (selfie) picture we use distinctive attributes of or cues in the picture to reach a personality judgment about the author of the selfie.

In Study 1 we tried to extract the observable characteristics of selfie pictures that can be helpful for observers in understanding the message of the picture. Since a selfie is photograph, we conducted qualitative analysis of its content according to the elements of composition, which is a fundamental characteristic of every photo. In a selfie picture, composition it is not subordinated to the skill of artistic expression, because a selfie by nature is a casual snapshot, thus, the composition reflects its author's habits, adopted social norms of visual expression, skills of sensorimotor coordination and conscious and unconscious personality characteristics (Frosh, 2015). The results of the Study 1 provided insight into the overall structure of the composition of selfies.

In Study 2 observable cues or coding categories were related to selected psychological constructs. In this sense, the analysis of Study 2 refers to an important segment of the lens model, i.e., cue validity (Qiu et al., 2015), and therefore, to the validity of the cues or coding categories of the selfies.

Consequently, both studies merge to yield an interpretation of selfies as “structured pictures that potentially reflect”; the coding scheme of selfie pictures is thus central and crucial for any further analysis and interpretation.

From the analysis of measures of intererater reliability (Study 1), we can conclude that one group of coding categories in the selfie coding scheme is intuitive and user-friendly, such as *eye contact, context, social distance*, and *tilt of the head*. The other group of coding categories has potential significance for inferences in the personality, but additional computer based accessories, such as lines and diagonals in the picture, improved the use of these cues. In this group are the categories *tilt of the body, tilt of the head*, and *head position*. *Camera position* is the most problematic category for coding, and the lack of contextualized cues from the process of selfie making probably implies that it cannot be objectively and reliably coded from the perspective of an independent coder. *One* important implication from both studies in the research is that it is reasonable to include categories with only a few sub-categories in the process of selfie coding.

Based on the identified cues or categories from the coding, we latter identified some basic characteristics of male and female selfies, relations to the context of the selfie pictures and relations with psychological constructs.

Comparatively, male selfies were centered in the picture; generally, the head was positioned around the center of the picture with very few expressions or body/head positions. In that sense, female selfies were less homogeneous and generally more expressive in the matter of head position, exhibition of the face, tilting of the head and mood expression.

Döring et al. (2016) found that the biggest difference between male and female selfies involved feminine touch and imbalance, which refers to canting of the head or body. Head canting is described as a gesture of submission (Key, 1975). We found that women tilted their heads more than males, but the results showed no relation to authority.

In female selfie pictures there was much diversity in the focus of the face compared to male pictures. On the other hand, the assumption about the focus being on the left side of the face in the majority of the selfie pictures (Bruno and Bertamini, 2013; Bruno et al., 2015) was not confirmed.

According to Kress and van Leeuwen (2006), the participant's gaze (direct eye contact) demands that the viewer enter into relationship with him. Other gestures or expressions determine what kind of relationship the participant wants to establish. When someone is smiling, he or she wishes to engage in a relation of social affinity. Female pictures emphasize smiling and eye contact (Tifferet and Vilnai-Yavetz, 2014). We found consistent results, with females who are more prone to smile in selfies than males.

Concerning the availability of selfies to others, participants whose selfies were available on social networks had higher results for emotional stability then participants whose selfies were available only to an intimate circle of people.

## Conclusions

The initial impression from the findings of our research analysis might be that there are no statistically important features of selfie pictures in relation to the personality characteristics of their owners. The implication might be that applying selfies in the context of personality assessment of the authors comes with reasonable reservations.

However, as emphasized in the introduction and design of the studies, the basic issue in our research was what can be said about the personality of the author on the basis of a single auto-portrait picture. In this regard, our sample, with its small number of participants and very narrow age range, is an obstacle to any firm generalization.

However, the perspective highlighted in our research was that, as important as the picture (selfie) is, it is also a selected selfie which is published, and the act of selection contributes to the final result. Therefore, the editorial process is a crucial part of selfie making. Our subjects have intentionally submitted (having reflected on and chosen) an image that they consider a prototype of the concept of “the selfie.” In this way, our research is distinct from studies which harvest images from the web, where researchers usually rely on the hash tags #selfie, #me or similar, and thus do not encounter similar drawbacks to potential generalization.

Another limitation of our research involves the analysis of effects in selfies and the context of selfie making (e.g., handedness of participants). Effects could potentially influence some aspects of coding (e.g., categories of background, frame of the picture or mood), but this could be of particular importance especially for the context of selfie analysis as impression formation, which was outside of the scope of the present research. Handedness, on the other hand, could especially influence the interpretation of camera position cues. However, camera position as a cue proved to be problematic, since assessment of this cue did not reach acceptable values of interrater reliability, despite additional software support in coding. Therefore, we excluded this cue from further analysis.

In search of a possible answer it is reasonable to consider lessons learned from the use of projection techniques. In this context one single product of a subject (e.g., a picture) may represent a basis for initial interpretation, but in the final stage more accurate implications in forming a personality impression of the subject require inclusion of information from other sources. Other sources could include other images or the results of other modalities of observation of the subject and monitoring of individuals over time (Hammer, 1968). More accurate or valid interpretation therefore derives from the integration of diverse data sources, and thus the principle of convergence should be applied. In the case of selfies, other available information from social networks could be taken into consideration, thus representing the broader context of the selfie picture and implying additional personality-related information about its author.

In this respect, a coding scheme for selfies with relatively few and simple (possible dichotomized) coding categories can represent a valuable initial tool in personality assessment. The set of coding cues is not exhaustive, and there is additional room for possible refinements, especially with the inclusion of more subjective measures (e.g., effects, attraction of the selfie).

In this first step, the research presented in this paper considers testing some features of a selfie with regard to general personality characteristics. Although the research was inspired by projective techniques, its aim was not to develop techniques for diagnosing personality disorders. Future work could explore this path and test selfies with regard to some established diagnostic model of personality disorders, like the hybrid dimensional-categorical model of DSM 5 (American Psychiatric Association, 2013).

Lessons learned from our research can also be seen as a step toward a broader understanding of the selfie concept, which could subsequently contribute to more objective debate on the phenomenon and shatter the widespread, everyday, intuitive idea of its ascribed pathological nature.

## Author contributions

BM was leader of the research team in both studies, coordinating the concept of studies, implementation and dissemination. In the manuscript participated in all parts of IMRaD structure, thus also for final version. AP participated in the all phases of research (conceptualization, implementation, dissemination). In manuscript contributed to the part of Methods, Results, and Discussion. TR participated in the all phases of research (conceptualization, implementation, dissemination). In manuscript contributed especially to Introduction, Methods and Discussion. LK participated in implementation and dissemination phases. She took especially active role in Study 1. NB participated in the all phases of research (conceptualization, implementation, dissemination). In manuscript contributed to all parts of IMRAD structure, but especially in conceptualization of Introduction and Discussion.

### Conflict of interest statement

The authors declare that the research was conducted in the absence of any commercial or financial relationships that could be construed as a potential conflict of interest.
