# Corrigendum: What Is Seen Is Who You Are: Are Cues in Selfie Pictures Related to Personality Characteristics?

**DOI:** 10.3389/fpsyg.2017.00353

**Published:** 2017-03-07

**Authors:** Bojan Musil, Andrej Preglej, Tadevž Ropert, Lucia Klasinc, Nenad C. Babič

**Affiliations:** ^1^Department of Psychology, Faculty of Arts, University of MariborMaribor, Slovenia; ^2^Faculty of Civil Engineering, Construction IT Centre, University of MariborMaribor, Slovenia

**Keywords:** selfie, self-presentation, social media, selfie coding, personality assessment

In the original article, there was an error. The wrong reference was cited, Hall and Pennington (2013), was cited instead of Hall (1964). A correction has been made to the **Coding Section, Sub-Section Social Distance, first sentence of the fifth paragraph:**

According to Hall (1964; see also Kress and van Leeuwen, 2006), we coded six social distances.

The authors apologize for this error and state that this does not change the scientific conclusions of the article in any way.

## Conflict of interest statement

The authors declare that the research was conducted in the absence of any commercial or financial relationships that could be construed as a potential conflict of interest.
